# Rescue Stenting for Failed Mechanical Thrombectomy in Acute Ischemic Stroke: Systematic Review and Meta‐analysis

**DOI:** 10.1161/SVIN.123.000881

**Published:** 2023-05-17

**Authors:** Aaron Rodriguez‐Calienes, Juan Vivanco‐Suarez, Milagros Galecio‐Castillo, Joel M. Sequeiros, Cynthia B. Zevallos, Mudassir Farooqui, Fazeel Siddiqui, Santiago Ortega‐Gutierrez

**Affiliations:** ^1^ Department of Neurology University of Iowa Hospitals and Clinics Iowa City IA; ^2^ Neuroscience Clinical Effectiveness and Public Health Research Group Universidad Científica del Sur Lima Peru; ^3^ Department of Neurology University of Tennessee Health Science Center Memphis TN; ^4^ Department of Neurology Metro Health University of Michigan Wyoming MI; ^5^ Department of Neurology Neurosurgery & Radiology University of Iowa Hospitals and Clinics Iowa City IA

## Abstract

**Background:**

When mechanical thrombectomy (MT) fails to achieve successful reperfusion, rescue stenting (RS) has proven to be a feasible rescue therapy. However, the available evidence remains underpowered to assess clinical outcomes. We aimed to compare the safety and efficacy of RS versus routine medical management in patients with failed MT using an aggregated meta‐analysis.

**Methods:**

A systematic review was performed from inception to July 2022 of all studies using RS after failed MT. Outcomes of interest included a modified Rankin scale score of 0–2 at 90 days, successful reperfusion (modified Thrombolysis in Cerebral Infarction 2b–3) after RS and symptomatic intracranial hemorrhage. A random‐effects meta‐analysis between the RS and medical treatment arms was performed to calculate pooled odds ratios (OR) for each outcome. We assessed the certainty of evidence using the Grading of Recommendation, Assessment, Development, and Evaluation approach. Statistical heterogeneity across studies was assessed with I2 statistics.

**Results:**

A total of 12 studies included 1855 participants, 729 in the RS arm and 1126 in the medical treatment arm. The pooled results indicated that RS was associated with a significantly higher proportion of patients with a modified Rankin scale score of 0–2 at 90 days (RS: 41% versus 21%; OR,3.27; [95% CI 2.08–5.16]; I2=64%; moderate‐certainty evidence) and a decreased risk of mortality at 90 days (RS: 22.5% versus 33.8%; OR, 0.47; [95% CI 0.32–0.69]; I2=45%; low‐certainty evidence), compared with medical treatment after failed MT. The pooled rate of successful reperfusion after RS was 87% (95% CI 82–91; I2=57%; low‐certainty evidence). The rate of symptomatic intracranial hemorrhage did not differ between groups (RS: 8.5% versus 11.7%; OR, 0.85; [95% CI 0.59–1.20]; I2=7%; low‐certainty evidence).

**Conclusion:**

RS is a promising strategy for maximizing recovery in acute stroke patients after first line MT fails to achieve meaningful reperfusion. However, randomized trials using a standardized approach/technique and MT failure definition are warranted to confirm these results.

## Graphical abstract

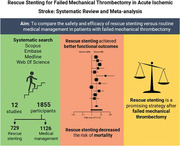

**Nonstandard Abbreviations and Acronyms**
IV‐tPA: intravenous tissue plasminogen activator
MM: medical management
mRS: modified Rankin scale
MT: mechanical thrombectomy
mTICI: modified Thrombolysis in Cerebral Infarction
RS: rescue stenting
sICH: symptomatic intracranial hemorrhage

**Clinical Perspective**

- In this meta‐analysis, we found better functional outcomes with rescue stenting in failed mechanical thrombectomy patients compared to medical management.
- We applicated robust measures to mitigate some of the risks involved with the retrospective studies meta‐analyzed.
- Due the lack of randomized controlled trial data, there has been ambiguity in the care of this specific patient population. Our findings help bridge this gap until well‐designed randomized control trials and prospective studies add power toward this field.

Approximately 10%–20% of patients treated with mechanical thrombectomy (MT) in clinical trials do not achieve successful reperfusion (modified Thrombolysis in Cerebral Infarction [mTICI] ≥2b).^1^, ^2^ This proportion might be higher in real‐world practice.^3^ Successful reperfusion is one of the strongest independent predictors for functional outcomes and mortality.^4^, ^5^, ^6^ In this setting, adjunctive medical treatment with antiplatelet glycoprotein IIb/IIIa inhibitors and/or endovascular treatment with intracranial stenting are considered rescue strategies.^7^

Recent data suggest that rescue stenting (RS) is associated with better functional outcomes when compared to leaving the vessel occluded or with inadequate reperfusion.^8^, ^9^, ^10^, ^11^ However, acute stent placement requires the use of antiplatelet therapy and/or angioplasty, which could potentially increase the risk of symptomatic intracranial hemorrhage (sICH). Of note, several recent publications evaluating RS on single‐arm cohorts^12^, ^13^, ^14^, ^15^, ^16^ and comparative studies^17^, ^18^, ^19^, ^20^, ^21^ evaluating patients with failed MT treated with RS versus medical management (MM) have shown similar safety between both approaches. Furthermore, recently published smaller systematic meta‐analyses using miscellaneous definitions of failed MT and moderate sample sizes also suggest that RS might be a safe and effective technique after failed MT.^11^, ^22^, ^23^, ^24^

Herein, we sought to evaluate the safety and efficacy of RS after failed MT and compare the clinical functional outcomes of patients with acute ischemic stroke treated with RS versus MM using a systematic review and meta‐analysis.

## Methods

### Protocol and Guidance

This systematic review used the Preferred Reporting Items for Systematic Reviews and Meta‐Analyses statement to report the search results.^25^ The protocol has been registered at the International Prospective Register of Systematic Reviews with the following registration code: CRD42022344534.

### Eligibility Criteria

The criteria for including studies in this review were the following: (1) randomized clinical trials, nonrandomized trials, and observational studies (≥10 participants); (2) patients with anterior or posterior circulation acute ischemic stroke with a failed MT (as defined by each study); (3) intracranial stent deployment used as a rescue technique following failed MT; and (4) studies that presented data from a group of patients managed with routine MM following failed MT. We excluded case reports, abstracts, posters, review articles, and studies in nonhumans.

### Information Sources and Search Strategy

Searches were performed in the following electronic databases: Scopus, Embase, Medline, and Web of Science from inception until July 2022. Likewise, a bibliographic search was carried out in external sources. The search strategy included Medical Subject Title terms for “stroke,” “thrombectomy,” and related words for “rescue stenting.” The full search strategy is provided in Supplementary Table S1. No language restrictions were considered for the selection of studies eligible for this review.

### Study Selection

The search strategy was applied individually to each database. All records were saved with EndNote X9 software and duplicates removed. Next, this record was exported in .XML format from EndNoteX9 to Mendeley to transform the data into RIS format to use the Rayyan tool (https://www.rayyan.ai/) for expert revision. Then duplicate articles were eliminated and 2 reviewers (A.R.C. and J.V.S.) screened the articles by titles and abstracts according to the inclusion criteria to identify potentially relevant articles. Any disagreements were resolved through initial discussion between the 2 reviewers and if no consensus was reached, a third reviewer was considered as an arbitrator (M.G.C.). When articles included overlapping populations, only the most relevant article with the largest population was selected.

### Data Collection Process

The data extracted included the following: year of publication, country, study design, single arm versus comparative study design, number of patients, past medical history (hypertension, diabetes, atrial fibrillation, hyperlipidemia, previous stroke, and smoking), baseline patient characteristics (baseline National Institutes of Health Stroke Scale, Alberta Stroke Program Early Computed Tomography Score), etiology of stroke, first‐line MT technique, number of MT attempts before RS, administration of intravenous tissue plasminogen activator (IV‐tPA), procedural characteristics (time metrics, occlusion location, type of stent employed, and use of concomitant balloon angioplasty), use of antiplatelets, stent reocclusion, and the prioritized outcomes.

### Outcomes and Prioritization

The primary efficacy outcome was the rate of functional independence according to the modified Rankin scale (mRS) at 90 days. A favorable functional outcome was defined by an mRS score of 0–2. The mRS is a categorical scale (0, no symptoms; 6, death) that reflects the degree of disability after a stroke. A score of 0–2 reflects independency for activities of daily living.^26^

Secondary efficacy outcomes were the rate of patients with successful reperfusion (mTICI 2b–3) after RS and the rate of patients with an excellent functional outcome (mRS 0–1). The mTICI is a categorical scale (range 0–3; the higher the number, the greater the degree of reperfusion) that reflects the degree of reperfusion and is scored on digital subtraction imaging.^27^

The primary safety outcome was the rate of sICH according to each study definition. The secondary safety outcome was the mortality rate at 90 days.

### Risk of Bias in Individual Studies

To assess the methodological quality of the studies included we used the risk of bias in nonrandomized studies of interventions for nonrandomized studies.^28^ We used the classification of low, moderate, serious, and critical risk of bias for nonrandomized studies of interventions following the instructions given in the Cochrane handbook for systematic reviews of interventions.^29^

### Data Synthesis

We used a random‐effects model in the meta‐analyses of studies providing data for both groups. We used the Mantel–Haenszel method to calculate odds ratio (OR) and 95% CIs for each outcome. For the meta‐analysis of proportions, we used a generalized linear mixed model for backward transformation and for obtaining pooled rates and 90% CIs. Additionally, we calculated prediction intervals based on t‐distribution.

We used the Cochran Q and I2 test for assessing statistical heterogeneity, with the following cutoffs: 0%–40%: might not be important; 30%–60%: may represent moderate heterogeneity; 50%–90%: may represent substantial heterogeneity; 75%–100%: considerable heterogeneity.^30^ Furthermore, we investigated sources of heterogeneity performing prespecified clinically relevant subgroup meta‐analyses by the stroke territory and the difference in stroke severity between arms. Studies with ≥2 points of difference in the mean/median admission National Institutes of Health Stroke Scale between arms were considered different in the stroke severity subgroup. In addition, we performed sensitivity analyses excluding studies with a low rate of IV‐tPA in the control arm, using a fixed‐effect model, excluding studies with a definition of failed MT not based on the mTICI, and only in studies with SR as the first‐line MT technique before RS. A *P* value for test for subgroup differences of <0.05 was considered significant. Analyses and plots were generated using R statistical software (version 4, 1.3) and R Studio.

### Meta‐Biases

If >10 studies were available for each outcome, we assessed publication bias by visual inspection of asymmetry in funnel plots.^31^ We also carried out Egger's tests.^32^

### Certainty of the Evidence

We assessed the certainty of the body of evidence of eligible studies in quantitative synthesis according to the Cochrane recommendations.^29^ We used the Grading of Recommendation, Assessment, Development, and Evaluation approach. This critical appraisal was based on considerations such as study design, risk of bias, inconsistency, indirectness, imprecision, and publication bias by Grading of Recommendation, Assessment, Development, and Evaluation.^33^ The certainty of the evidence was characterized as high, moderate, low, or very low and reported as a summary of findings table, performed in the Grading of Recommendation, Assessment, Development, and Evaluation online tool (http://gradepro.org).

## Results

### Study Selection

We identified a total of 6711 documents during the initial search and removed 3048 duplicates (Supplementary Figure S1). In the review by title and abstract there were 3663 potentially eligible documents. In the full‐text evaluation, we excluded 49 documents (Supplementary Table S2) and finally included 26 studies. Two studies included overlapping populations; therefore, we used one^12^ for the comparative meta‐analysis and the other^34^ for the single‐arm meta‐analysis.

### Study Characteristics

Twelve nonrandomized comparative cohorts involved 1855 participants, 729 in the RS arm and 1126 in the control arm (Table 1). Fourteen single‐arm cohorts included 684 patients treated with RS (Supplementary Table S3).

**Table 1:** Clinical and Treatment Characteristics of the Comparative Studies

Study	No. of patients		Age (y)		NIHSS		ASPECTS		IV‐tPA (%)		RS arm			
	RS	MM	RS	MM	RS	MM	RS	MM	RS	MM	First line technique	Definition of failed MT	No. of attempts before RS	Type of stent
Tschoe et al., 2022^21^	50	267	67 [61–75]	66 [56–76]	16 [12–20]	18 [14–22]	9 [8‐10]	9 [8–10]	58	91	–	mTICI 0–1	3 [2‐4]	–
Sweid et al., 2022^20^	34	94	66 (1.5)	70 (15.6)	14.2 (8.3)	16.5 (7.2)	–	–	29	39	SR, DA, Solumbra	mTICI 0–2a	2.6 (1.1)	Enterprise: 22; Wingspan: 4; Neuroform: 1; Atlas: 6
Mohammaden et al., 2022^19^	253	246	64.8 (13.8)	70 (15.7)	15 [10–19]	18 [12–22]	9 [8–10]	8 [7–9]	4	6	–	mTICI 0–1	2 [1–4]	–
Luo et al., 2021^18^	81	12	61.1 (11.9)	63 (13.4)	23	20	7	7	14	33	SR: 74; DA: 2	High‐grade stenosis and immediate reocclusion	2 (1)	–
Hassan et al., 2021^17^	46	43	70.3 (13.8)	71 (12.9)	16.2 (7.9)	16.4 (8.2)	–	–	15	40	–	mTICI 0–2a	–	–
Pérez‐Garcíaet al., 2020^40^	20	40	61.8 (16.5)	69.5 (13)	18 [8–28]	20 [12–29]	8 [6–10]	8 [5–11]	35	38	SAVE technique	mTICI 0–1	3.5 (2.1)	Solitaire: 3; Acclino flex: 1; Enterprise: 3; Wingspan: 9; Neuroform: 6; Atlas: 2
Peng et al., 2020^39^	90	117	65 [52–75]	69 [59–76]	15 [14–21]	18 [14–22]	9 [8‐10]	9 [8–9]	30	32	SR: 90	mTICI 0–2a	2 [1–4]	Solitaire: 70; Apollo: 18
Cornelissen et al., 2019^10^	12	14	65.2 (13.9)	67.3 (9.5)	17 [5–22]	17 [8–22]	–	–	25	43	SR: 12	mTICI 0–1	3.5 [1–8]	Solitaire: 4; Enterprise: 8
Zhou et al., 2018^41^	47	146	62.2 (13.4)	63 (11.8)	–	–	–	–	26	23	SR: 47	mTICI 0–2a	–	Solitaire: 24; Apollo: 16; Enterprise: 5; Wingspan: 6; Neuroform: 4
Baracchini et al., 2017^38^	23	23	70 (16.9)	74 (8.3)	16 [4–26]	18 [5–20]	–	–	17	43	SR: 23	Reocclusion after MT	–	Solitaire: 23
Chang et al., 2018^9^	48	100	63.7 (16.8)	68 (12.1)	14 [6–22]	15 [9–21]	8 [6–9.8]	8 [6–10]	46	46	SR: 36; DA: 3; combined: 9	mTICI 0–2a	3.4 (1.6)	Solitaire: 37; Enterprise: 2; Wingspan: 8
Baek et al., 2021^34^	25	24	69.7 (13.2)	75 (11.9)	17 [16–18]	16 [12–19]	8 [7–9]	7.5 [6–9]	20	46	SR: 18; DA: 7	mTICI 0–2a	3.2 (2)	–

ASPECTS indicates Alberta Stroke Program Early Computed Tomography Score; DA, direct aspiration; IV‐tPA, intravenous tissue plasminogen activator; MC, multicentric; MM, medical management; MT, mechanical thrombectomy; mTICI, modified Thrombolysis in Cerebral Infarction; NIHSS, National Institutes of Health Stroke Scale; No, number; RS, rescue stenting; SC, single‐center; and SR, stent retriever.

In the pooled population of RS patients (1413 patients), the mean age ranged from 61 to 73 years, and 36% of the patients were female. Hypertension, diabetes, atrial fibrillation, hyperlipidemia, previous stroke/transitory ischemic attack, and smoking were present in 66%, 30%, 17%, 33%, 25%, and 29%, respectively. Additional details regarding stroke territory and time metrics are presented in Supplementary Table S4. The median National Institutes of Health Stroke Scale score ranged from 4.5 to 22 and the median Alberta Stroke Program Early Computed Tomography Score ranged from 7 to 9. The overall use of IV‐tPA was 26%. First‐line treatment with stent retriever was documented in 84% of the cases. A protocol of an IV bolus of a glycoprotein IIb/IIIa inhibitor was used in 14 studies with a maintenance infusion reported in 9 studies (Supplementary Table S5). Most of the studies (16/26) defined failed MT as unsuccessful reperfusion based on the mTICI (mTICI 0–1 or mTICI 0–2a) from the first‐line treatment. The median number of attempts before RS ranged from 2 to 4. The detachable Solitaire stent was the most commonly deployed permanent stent (42%).

### Risk of Bias Among Studies

The overall risk of bias was serious among studies (Supplementary Figure S2). Most of the studies (84%) were retrospective in nature, which results in selection bias and lack of control for confounding. There were no deviations from the intendent intervention in the studies. Five studies^14^, ^16^, ^35^, ^36^, ^37^ were assessed as at serious risk of bias because they reported missing data for the prioritized outcomes of this meta‐analysis. Approximately half of the studies (46%) were multicentric without a predefined decision algorithm to select patients for RS or a core laboratory; this leads to between‐center heterogeneity and a serious risk of bias in the measurement of outcomes.

Publication bias was explored by inspecting the funnel plots. Egger's tests were not suggestive of publication bias (Supplementary Table S6).

### Synthesis of Results

The effects of interventions with the certainty assessment are presented on the summary of findings table (Table 2).

**Table 2:** Summary of Findings of Medical Management Versus Rescue Stenting Therapies

Outcomes	Anticipated absolute effects (95% CI)		Relative effect (95% CI)	No of participants (studies)	Certainty of the evidence (GRADE)
	Medical management	Rescue stenting			
Favorable functional outcome follow‐up: 90 days	Study population		OR 3.27 (2.08–5.16)	1773 (12 observational studies)	⊕ ⊕ ⊕ ◯ Moderate^†^, ^‡^
	212 per 1000	467 per 1000 (358–581)			
Successful reperfusion	*		0.87** (0.82–0.91)	1412 (25 observational studies)	⊕ ⊕ ◯◯ Low^‡^, ^¶^, ^§^
	*	*			
Symptomatic intracranial hemorrhage	Study population		OR 0.85 (0.59–1.20)	1854 (12 observational studies)	⊕ ⊕ ◯◯ Low^†^, ^||^
	107 per 1000	92 per 1000 (66–125)			
Mortality follow‐up: 90 days	Study population		OR 0.47 (0.32–0.69)	1711 (11 observational studies)	⊕ ⊕ ◯◯ Low^†^, ^#^
	338 per 1000	194 per 1000 (140–261)			

GRADE indicates Grading of Recommendation, Assessment, Development, and Evaluation; OR, odds ratio; and ROBINS–I, risk of bias in non‐randomized studies of interventions. ^*^Not calculable. ^**^Pooled rate.

^†^ Based on the ROBINS‐I quality assessment.

^‡^ I^2^ = 64%.

^¶^ I^2^ = 57%.

^§^ Not comparative analysis with medical treatment.

^||^ Based on the CI of our results.

^#^ I^2^ = 45%.

**Figure 1:**
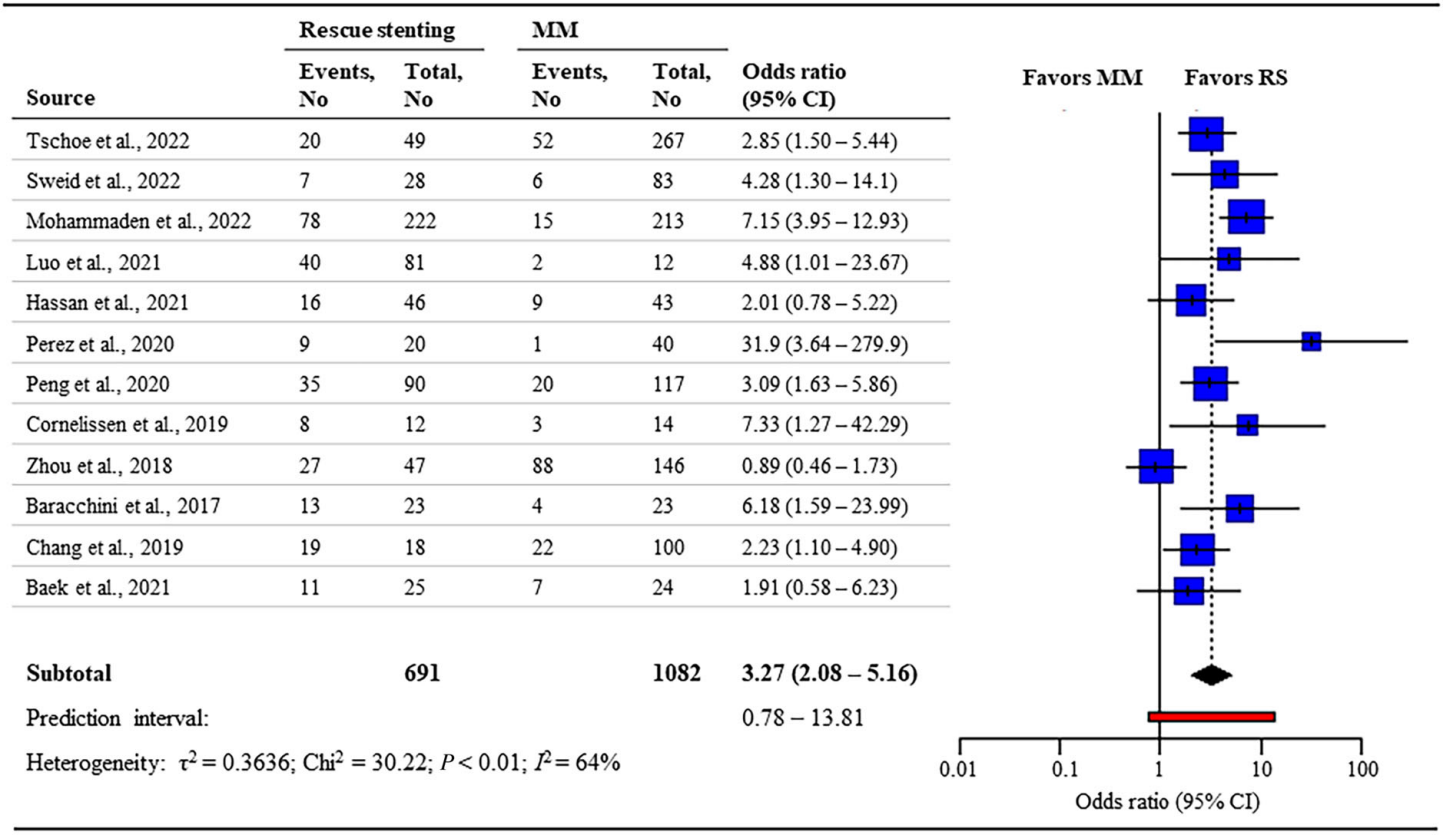
**Forest plot for rescue stenting versus medical management for favorable functional outcome defined as modified Rankin scale (mRS)≤2**. MM indicates medical management; and RS, rescue stenting.

Data for favorable functional outcomes were available in a total of 1773 participants from 12 studies.^9^, ^10^, ^17^, ^18^, ^19^, ^20^, ^21^, ^34^, ^38^, ^39^, ^40^, ^41^ Patients treated with RS had higher odds of achieving a favorable functional outcome (41% versus 21.1%; OR, 3.27; [95% CI 2.08–5.16]; moderate‐certainty evidence) with substantial between‐study heterogeneity (I2=64%) (Figure 1). A sensitivity analysis excluding studies with low rates (<40%) of IV‐tPA treatment in the medical management arm yielded similar results (45% versus 21%; OR, 2.81; [95% CI 1.94–4.08]) with little between‐study heterogeneity (I2=0%). Furthermore, the sensitivity analysis using a fixed‐effect model found similar results compared to the random‐effect model (Supplementary Table S7).

In relation to successful reperfusion, data were available in a total of 1412 patients treated with RS from 25 studies.^9^, ^10^, ^12^, ^13^, ^14^, ^15^, ^16^, ^17^, ^18^, ^19^, ^20^, ^21^, ^35^, ^36^, ^37^, ^38^, ^39^, ^40^, ^41^, ^42^, ^43^, ^44^, ^45^, ^46^, ^47^ The rate of successful reperfusion after RS was 87% (95% CI 82%–91%; low‐certainty evidence) with substantial between‐study heterogeneity (I2=57%). A sensitivity analysis excluding studies that used a definition of failed MT not based on the initial mTICI found reperfusion rates of 85% (95% CI 80%–89%; I2=67%) (Supplementary Table S7).

**Figure 2:**
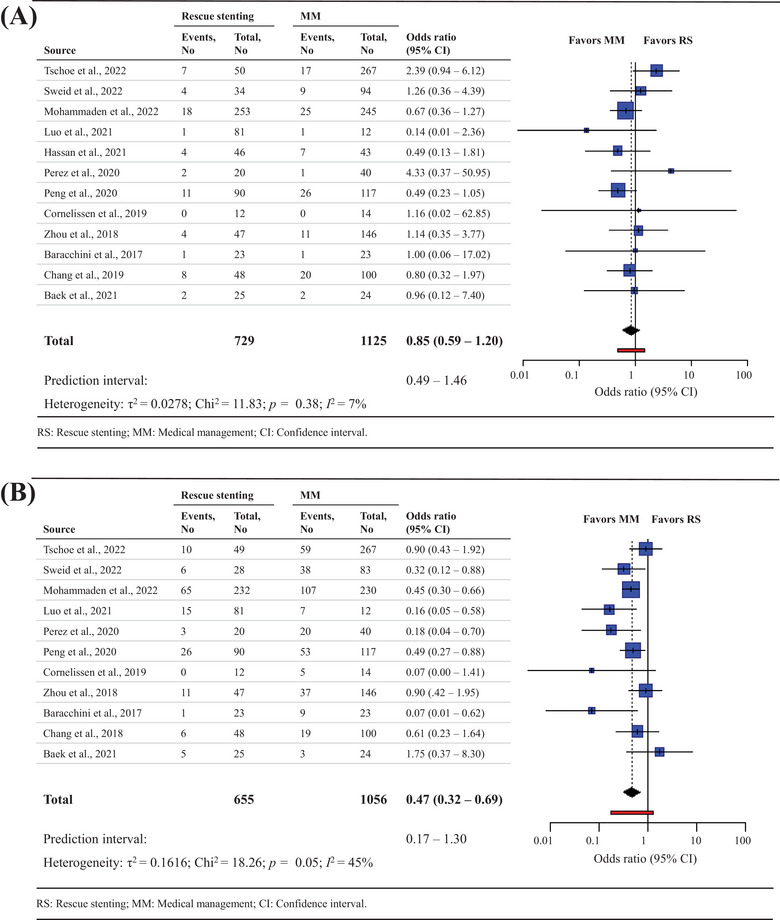
**Forest plot for rescue stenting (RS) versus medical management for (A) symptomatic intracranial hemorrhage (sICH) and (B) mortality at 90 days**. MM indicates medical management.

Data on sICH were available in a total of 1854 participants from 12 studies.^9^, ^10^, ^17^, ^18^, ^19^, ^20^, ^21^, ^34^, ^38^, ^39^, ^40^, ^41^ There were no differences between the RS arm and the medical treatment arm in sICH (8.5% versus 11.7%; OR, 0.85; [95% CI 0.59–1.20]; low‐certainty evidence) with very little between‐study heterogeneity (I2=7%) (Figure 2A). A sensitivity analysis of studies in which stent retriever was the exclusive first‐line MT technique before RS yielded similar results (9% versus 13%; OR, 0.65; [95% CI 0.35–1.21]) with little between‐study heterogeneity (I2=0%) (Supplementary Table S7).

For mortality at 90 days, data were available in a total of 1711 participants from 11 studies. ^9^, ^10^, ^18^, ^19^, ^20^, ^21^, ^34^, ^38^, ^39^, ^40^, ^41^ Compared with medical treatment RS resulted in a reduced risk of mortality at 90 days (22.5% versus 33.8%; OR, 0.47; [95% CI 0.32–0.69]; low‐certainty evidence) with moderate between‐study heterogeneity (I2=45%) (Figure 2B). A sensitivity analysis excluding studies with low rates (<40%) of IV‐tPA treatment in the medical management arm resulted in loss of the reduced risk of mortality with RS (16% versus 23%; OR, 0.42; [95% CI 0.17–1.03]) with substantial between‐study heterogeneity (I2=61%) (Supplementary Table S7).

### Subgroup Analysis

Subgroup analysis between studies that included only anterior circulation stroke versus studies with anterior and posterior circulation stroke for favorable functional outcomes, successful reperfusion, sICH, and mortality at 90 days was not significant (Supplementary Figure S3).

Subgroup analysis for studies with different stroke severity between arms versus without different stroke severity between arms for favorable functional outcomes and mortality at 90 days was not significant between subgroups (Supplementary Figure S4).

## Discussion

Evidence from our meta‐analysis of patients with acute ischemic stroke and refractory initial MT therapy suggest that (1) RS increases the likelihood of a favorable functional outcome at 90 days compared to MM after failed MT, with moderate‐certainty evidence; and (2) RS after failed MT does not increase the risk of sICH, with low‐certainty evidence. In addition, low‐certainty evidence suggests that RS can achieve high rates of successful reperfusion after failed MT.

Our meta‐analysis including 1773 patients confirmed the superior efficacy of RS over routine MM after failed MT with a favorable outcome (90‐day mRS 0–2) pooled rate of 41% after RS. This finding agrees with 3 previously published smaller sized meta‐analyses with miscellaneous definitions of failed MT.^11^, ^22^, ^24^ Almallouhi et al. included 507 patients from 5 comparative studies and defined failed MT based on the presence of intracranial atherosclerosis.^24^ In the study by Premat et al., which included 352 patients from 4 comparative studies, the definition of failed MT was based on the number of MT attempts.^11^ Finally, in the study by Cai et al., which included 704 patients from 7 comparative studies, a failed MT was based on the final mTICI score^22^ (Supplementary Table S8). Furthermore, our rates are quite similar to the functional outcome rates of the HERMES (Highly Effective Reperfusion Using Multiple Endovascular Devices; 46%)^2^ and AURORA (Analysis of Pooled Data From Randomized Studies of Thrombectomy More Than 6 Hours After Last Known Well; 45.9%) studies,^48^ both of which were patient‐level meta‐analyses in which MT was successfully performed in 71% and 81% of cases, respectively. Given the observational design of the studies analyzed, it is reasonable to consider the existence of several imbalanced confounding factors that might have influenced clinical outcomes after RS. Some of these factors are baseline stroke severity, etiology of the stroke (internal carotid artery disease, embolism), individual collateral status, time to reperfusion, periprocedural use of antithrombotics, and the development of intracranial hemorrhage.^12^ In an attempt to optimize the comparison with the control group, we performed a sensitivity analysis excluding the studies with low rates of IV‐tPA treatment. In our subgroup analyses, we were able to account for baseline stroke severity and the anatomical territory of the stroke. The effect size of RS on clinical outcomes remained unchanged in all the subgroup and sensitivity analyses.

In our study, the successful pooled reperfusion rate in patients with RS after failed MT was 87%. This finding is also comparable to the reperfusion rate of a recent meta‐analysis by Cai et al. (82%)^22^ and help to emphasize the concept that final reperfusion status remains a key predictor of favorable outcome regardless of the technique.^4^, ^5^, ^6^ Moreover, it confirms that reperfusion of the occluded vessel can be achieve with a variety of self‐expanding or balloon‐mounted stents after initial MT attempts have failed. Finally, timely reperfusion done safely may be the critical factor that primarily affects neurological recovery.

Our pooled sICH rates were similar in the RS and MM groups (8.5% and 11.7%) and were also similar to other studies including a retrospective registry (10%),^21^ a real‐world registry (8% in the early time window, and 10.9% within 6–16 hours and 5% within 16–24 hours),^49^ and a single‐arm meta‐analysis of patients who underwent expandable stents following failed anterior circulation thrombectomy (11.6%).^23^ However, the sICH rates were still higher than the randomized controlled trials interventional groups by the HERMES (4.4%),^2^ and AURORA (5.3%) patient data meta‐analyses.^48^ Longer procedural times and multiple devices passes are associated with sICH.^50^, ^51^ In our groups, the median number of attempts before RS ranged from 2 to 4. Other probable risk factors for an increased sICH rate are the use of IV‐tPA, antiplatelet glycoprotein IIb/IIIa inhibitors, and subsequent dual antiplatelet therapy.^47^, ^52^ In our analysis, the overall use of IV‐tPA was 26%; most of the studies included did not report if they used IV antiplatelet therapy. Other variables such as uncontrolled hypertension, glucose and baseline and final infarct volume after reperfusion are also associated with increased rates of sICH^53^ but were not collected in the majority of the studies included.

Unsuccessful revascularization is an independent predictor for mortality^5^, ^6^ and, therefore, we expected a lower mortality in the RS group compared with MM (22.5% versus 33.8%), with this proportion being comparable with other single‐arm meta‐analyses (39%^11^, ^23^).The higher mortality rate in the MM group was consistent with a multivariate regression analysis showing that unsuccessful reperfusion is the strongest predictor of mortality in patients undergoing MT.^6^

With the increasing awareness and expansion of MT, there is a need for large prospective studies and/or randomized trials to prove the efficacy of RS in patients with failed MT, knowing that without any rescue interventions, these patients carry higher risk for sICH, mortality, and disability.^54^, ^55^ In our study, RS showed better functional independence and mortality with similar rates of sICH and could be considered as a tier potential treatment for this population who otherwise does not have other options. To avoid longer procedural time and multiple passes, it is critical to define MT failure to provide early rescue therapy; however, an evidence‐based definition is still lacking. More than 90 minutes of procedure or ≥3 passes were the most used in the studies included in our analysis. It is also of greater interest to identify reliable prethrombectomy imaging markers to identify these patients before offering MT, because this would be helpful in reducing selection bias as well as using antiplatelet therapy in anticipation for possible RS. Our group and others have studied imaging biomarkers that might be helpful to consider in the context of an initial failed MT. These markers include absent hyperdense sign on noncontrast computed tomography head at the site of occlusion, quantitative measurements of hyperdensity ipsilateral and contralateral to the site of occlusion, and truncal type of occlusion on computed tomography angiography or catheter‐based angiography have shown promise.^56^, ^57^, ^58^, ^59^, ^60^, ^61^

### Limitations

The most severe constraint of this meta‐analysis was the quality of the studies included. All were observational and most were single center and retrospective. Therefore, confounding factors were not controlled and could produce heterogeneity and affect the outcomes reported. Methodological heterogeneity was observed among studies in the definition of failed MT, the criteria for proceeding with RS, the treatment approaches, the types of stents used, the number of MT attempts, adjunctive balloon angioplasty, and antiaggregation therapy. Furthermore, considering that the etiology of the occlusion differs among populations and that intracranial atherosclerotic‐related occlusion is refractory to stent retrievers and is associated with lower reperfusion rates and higher reocclusion rates when compared with other etiologies,^62^ the lack of information about this variable is a considerable source of confounding. In addition, information about potential complications of RS, such as vessel perforation, rupture, and stent reocclusion, were not available. Therefore, with the aim to address confounding factors and other biases, we performed sensitivity and subgroup meta‐analyses with different approaches for defining study eligibility criteria (Supplementary Table S7) and qualitative methods (risk of bias in nonrandomized studies of interventions tool [Supplementary Figure S2] and the Grading of Recommendation, Assessment, Development, and Evaluation assessment of certainty [Table 2]) to address the methodological issues that could affect the validity of our results, as recommended when meta‐analyzing nonrandomized studies.^63^

In this meta‐analysis, we included studies with posterior circulation stroke patients. The use of the conventional stroke assessment scales (mRS, mTICI, and National Institutes of Health Stroke Scale) may not adequately measure the patient's clinical deficits and response to treatment. Finally, long‐term outcomes of RS, such as the arterial patency, recurrence of stroke, and functional independence, were not studied.

## Conclusion

RS is a promising strategy for maximizing recovery in acute stroke patients after first‐line MT fails to achieve meaningful reperfusion. Clinically available high predictive biomarkers that identify patients that will likely fail MT present in noninvasive neuroimaging represent a critical initial first step. Then, randomized trials using a standardized approach/technique and independently adjudicated outcomes are warranted to confirm this observation.

## Sources of Funding

None.

## Disclosures

Ortega‐Gutierrez – Grants: NIH‐NINDS (R01NS127114‐01), Stryker, Medtronic, Microvention, Methinks, IschemiaView, Viz.ai, Siemens. Consulting fees: Medtronic, Stryker Neurovascular.

## Supporting information

Supplementary Information
