# Supplementary material for: Rescue Stenting for Failed Mechanical Thrombectomy in Acute Ischemic Stroke: Systematic Review and Meta‐analysis
Source: Stroke Vasc Interv Neurol. 2023 May 17;3(4):e000881. doi: 10.1161/SVIN.123.000881 (PMC12778681; doi:10.1161/SVIN.123.000881)
Supplement: Supplementary file 1 — Supplementary Information [file SVI2-3-e000881-s001.pdf]

SUPPLEMENTAL MATERIALS

Rescue Stenting for Failed Mechanical Thrombectomy in Acute Ischemic Stroke: Systematic Review and Meta-analysis

Authors

Aaron Rodriguez-Calienes[1,2], MD; Juan Vivanco-Suarez[1], MD; Milagros Galecio-Castillo[1], MD; Joel M Sequeiros[3], MD; Cynthia B. Zevallos[1], MD; Mudassir Farooqui[1], MD; Fazeel Siddiqui[4], MD; Santiago Ortega-Gutierrez[5], MD, MSc

[1] Department of Neurology, University of Iowa Hospitals and Clinics, Iowa City, IA, USA
[2] Neuroscience, Clinical Effectiveness and Public Health Research Group, Universidad Científica del Sur, Lima, Peru
[3] Department of Neurology, University of Tennessee Health Science Center, Memphis, Tennessee, USA
[4] Department of Neurology, Metro Health University of Michigan, Wyoming, MI

[5]Department of Neurology, Neurosurgery & Radiology, University of Iowa Hospitals and Clinics, Iowa City, IA, USA

Supplementary Table 1. Search strategy for each database.

MEDLINE		
**Step**	**Search query**	**Results**
#1	"Stroke"[Mesh] OR Stroke[TIAB] OR (large[tiab] AND Occlusion[tiab]) OR LVO[tiab]	2418
#2	(rescue[tiab] AND (therapy[tiab] OR treatment[tiab] OR technique[tiab])) OR (rescue[tiab] AND ("Stents"[Mesh] OR stent*[tiab]) OR stent*[tiab] OR “intracranial stenting”[tiab]	
#3	((fail*[tiab] OR unsuccessful[tiab] OR refractory[tiab] OR resist*[tiab]) AND (“thrombectomy”[tiab] OR “mechanical thrombectomy”[tiab] OR endovascular[tiab] OR reperfusion[tiab])) OR (“thrombectomy”[tiab] OR Thrombectomy[MH])	
	((#1 AND #2) AND #3)	

Scopus		
**Step**	**Search query**	**Results**
#1	INDEXTERMS(“Stroke”) OR TITLE-ABS(Stroke) OR (TITLE-ABS(large) AND TITLE-ABS(Occlusion) OR TITLE-ABS(LVO))	1713
#2	(TITLE-ABS(rescue) AND (TITLE-ABS(therapy) OR TITLE-ABS(treatment) OR TITLE-ABS(technique))) OR (TITLE-ABS(rescue) AND (INDEXTERMS(“Stents”) OR TITLE-ABS(stent))) OR TITLE-ABS(stent) OR TITLE-ABS(“intracranial stenting”)	
#3	((TITLE-ABS(fail) OR TITLE-ABS(unsuccessful) OR TITLE-ABS(refractory) OR TITLE-ABS(resist)) AND (TITLE-ABS(“thrombectomy”) OR TITLE-ABS(“mechanical thrombectomy”) OR TITLE-ABS(endovascular) OR TITLE-ABS(reperfusion))) OR (TITLE-ABS(“thrombectomy”) OR INDEXTERMS(Thrombectomy))	
	((#1 AND #2) AND #3)	

EMBASE		
**Step**	**Search query**	**Results**
#1	stroke/exp OR (large:ti,ab AND occlusion:ti,ab) OR lvo:ti,ab	1716
#2	(rescue:ti,ab AND (therapy:ti,ab OR treatment:ti,ab OR technique:ti,ab)) OR (rescue:ti,ab AND (stents/exp OR stent:ti,ab)) OR stent:ti,ab OR 'intracranial stenting':ti,ab	
#3	((fail:ti,ab OR unsuccessful:ti,ab OR refractory:ti,ab OR resist:ti,ab) AND (thrombectomy:ti,ab OR 'mechanical thrombectomy':ti,ab OR endovascular:ti,ab OR reperfusion:ti,ab)) OR (thrombectomy:ti,ab AND thrombectomy/exp)	
	((#1 AND #2) AND #3) OR #4	

Web of Science		
**Step**	**Search query**	**Results**
#1	TS=("Stroke")	863
#2	(TS=(Rescue) AND TS=(Therap* OR treatment OR technique)) OR TS=(stent*)	
#3	(TS=(thrombectomy OR endovascular OR reperfusion OR “mechanical thrombectomy”) AND TS=(fail* OR unsuccessful OR resist* OR refractory)) OR TS=(Failure w/5 thrombectomy)	
#4	#2 AND #3	
#5	#1 AND #4	

Supplementary Table 2. Table of studies excluded.

Author	Title	Reason of exclusion
Yoon W et al.	Acute basilar artery occlusion-Difference in characteristics and outcomes after mechanical thrombectomy between patients with and without underlying severe intracranial atherosclerotic stenosis	Wrong publication type
Rho M et al.	Acute reperfusion treatment mechanical thrombectomy with solitaire stent and forced arterial suction thrombectomy (fast) with penumbra reperfusion catheter: Which device is fast and effective?	Paper not available
Kurisu et al.	ADAPT First-Line Strategy for MCA Mainstem Occlusion; Analysis for Optimal Salvage Therapy and its Related Factor	Wrong population
Seo JH et al.	Adjuvant Tirofiban Injection Through Deployed Solitaire Stent As a Rescue Technique After failed Mechanical Thrombectomy in Acute Stroke.	Wrong intervention
Lu J. et al	Analysis of intracranial cerebral hemorrhage in patients after receiving rescue thrombectomy with Solitaire AB stent for acute ischemic stroke	Paper not available
Chen R. et al	Analysis of reperfusion techniques for acute large vessel occlusion caused by intracranial atherosclerotic stenosis	Paper not available
Wu C et al	Angioplasty and/or stenting after thrombectomy in patients with underlying intracranial atherosclerotic stenosis.	Wrong intervention
Zhou T-F et al	Application of Solitaire AB stent in endovascular treatment of acute ischemic stroke	Wrong outcome
Chen F et al	A Prospective, Multicenter, Single-Group Target-Value Clinical Trial to Evaluate the Safety and Efficacy of a Large Bore Aspiration Catheter System for the Endovascular Treatment of Acute Ischemic Stroke.	Wrong population
Limbucci N et al	Bail out intracranial stenting with after unsuccessful thrombectomy	Paper not available
Negida et al	Can the adapt technique be advocated over the standard stentretriever technique for acute stroke patients with a large vessel occlusion a meta-analysis of published literature	Wrong study design
Kurre W et al	Clinical experience with the pREset stent retriever for the treatment of acute ischemic stroke - A review of 271 consecutive cases	Wrong outcome
Requena et al	Clinical Results of the Advanced Neurovascular Access Catheter System Combined With a Stent Retriever in Acute Ischemic Stroke (SOLONDA).	Wrong population
Hesse AC et al	Comparison of radiological outcomes in patients with acute ischemic stroke treated with different thrombectomy techniques	Paper not available
Kim SH et al	Differences between proximal and distal M1 occlusions after mechanical thrombectomy.	Wrong population
Zhang G et al	Direct angioplasty for acute ischemic stroke due to intracranial atherosclerotic stenosis-related large vessel occlusion	Wrong population
Patro SN et al	Dual-stent retrieval for mechanical thrombectomy of refractory clot in acute stroke as a rescue technique	Wrong publication type
Fujiwara S et al	Dual-stent retrieval for mechanical thrombectomy of refractory clot in acute stroke as a rescue technique	Wrong publication type
Li W et al	Emergency Angioplasty or Stenting for Stroke Patients with Intracranial Atherosclerotic Large Vessel Occlusion	Wrong population
Fan Y et al	Emergency Angioplasty or Stenting for Stroke Patients with Intracranial Atherosclerotic Large Vessel Occlusion	Wrong population
Tsang et al	Endovascular thrombectomy for occlusions due to intracranial atherosclerosis: Correlation with intracranial carotid artery calcification and treatment implications	Wrong population
Park H et al	Endovascular Treatment of Acute Stroke Due to Intracranial Atherosclerotic Stenosis-Related Large Vessel Occlusion	Wrong publication type
Stracke et al	Intracranial bailout stenting with the Acclino (Flex) Stent/NeuroSpeed Balloon Catheter after failed thrombectomy in acute ischemic stroke: A multicenter experience	Overlapping population
Forbrig R et al	Intracranial rescue stenting after failed Stent-retriever thrombectomy due to an underlying lesion	Overlapping population
Jia B et al	Mechanical thrombectomy and rescue therapy for intracranial large artery occlusion with underlying atherosclerosis.	Wrong outcome
Sang et al	Mechanical Thrombectomy Using Solitaire in Acute Ischemic Stroke Patients with Vertebrobasilar Occlusion: A Prospective Observational Study	Wrong outcome
Perry DaCamara C et al	Middle cerebral artery stenting in hyperacute ischemic stroke: Single center experience	Wrong publication type
Kang DH et al	Need for rescue treatment and its implication: Stent retriever versus contact aspiration thrombectomy	Wrong intervention
Stapleton et al	Noninferiority of a direct aspiration first-pass technique vs stent retriever thrombectomy in emergent large-vessel intracranial occlusions	Wrong publication type
Baek et al	Outcomes of endovascular treatment for acute intracranial atherosclerosis-related large vessel occlusion	Overlapping population
Al Kasab S et al	Outcomes of rescue endovascular treatment of acute ischemic stroke in patients with underlying intracranial atherosclerosis-insights from star registry	Wrong publication type
Al Kasab S et al	Outcomes of Rescue Endovascular Treatment of Emergent Large Vessel Occlusion in Patients With Underlying Intracranial Atherosclerosis: Insights From STAR.	Wrong publication type

Ahmed SU et al	Permanent implantation of the Solitaire device as a bailout technique for large vessel intracranial occlusions	Wrong population
Arquizan C et al	Rescue, combined and standalone thrombectomy for stroke management in large vessel occlusion using the Solitaire™ FR device. A prospective single center study. (Recost study)	Wrong publication type
Costalat V et al	Rescue, Combined, and Stand-Alone Thrombectomy in the Management of Large Vessel Occlusion Stroke Using the Solitaire Device: A Prospective 50-Patient Single-Center Study Timing, Safety, and Efficacy	Wrong intervention
Khatibi K et al	Rescue intracranial angioplasty with or without stenting in acute ischemic stroke	Wrong publication type
Bhatti et al	Rescue Strategies in Anterior Circulation Stroke with Failed Mechanical Thrombectomy-A Retrospective Observational Study (RAFT)	Wrong population
Tsang et al	Severity of intracranial carotid artery calcification in intracranial atherosclerosis-related occlusion treated with endovascular thrombectomy.	Wrong population
Daou BJ et al	Stent Placement in Mechanical Thrombectomy for Acute Ischemic Stroke: A Multi-Institutional Experience	Wrong publication type
Tonetti DA et al	Stentriever salvage after failed manual aspiration thrombectomy	Wrong population
Ni CF et al	Added Value of Rescue Devices in Intra-Arterial Thrombectomy: When Should We Apply Them?	Wrong intervention
Stapleton et al	A direct aspiration first-pass technique vs stentriever thrombectomy in emergent large vessel intracranial occlusions	Wrong intervention
Aydin et al	Crossing Y-Solitaire thrombectomy as a rescue treatment for refractory acute occlusions of the middle cerebral artery	Wrong intervention
Cabral et al	Device size selection can enhance Y-stentrieving efficacy and safety as a rescue strategy in stroke thrombectomy	Wrong intervention
Akmangit I et al	Endovascular Stroke Therapy Focused on Direct Clot Aspiration Using Sofia Catheter in Acute Ischemic Stroke	Wrong intervention
Zhou T et al	Rescue stent implantation for the treatment of acute ischemic stroke: Initial experience in 13 patients	Overlapping population
Piasecki P et al	Safety and Efficacy of Mechanical Thrombectomy Using Tigertriever as a Rescue Device After Failed Aspiration-Single Center Experience.	Wrong intervention
Baek JH et al	Stenting as a Rescue Treatment After Failure of Mechanical Thrombectomy for Anterior Circulation Large Artery Occlusion.	Overlapping population
Li ZF et al	Y-Stent Rescue Technique for Failed Thrombectomy in Patients With Large Vessel Occlusion: A Case Series and Pooled Analysis	Wrong intervention

Supplementary Table 3. Clinical and treatment characteristics of the single-arm rescue stenting studies.

Study	Design	Clinical characteristics					Treatment characteristics				
		N	Age (years)	Female (%)	NIHSS	ASPECTS	IV-tPA (%)	First line technique	Definition of failed MT	Attempts before RS	Type of stent
Baek et al., 2022 [12]	Prospective, MC	78	64.6 (14.2)	35	13 [9-18]	8 [7-9]	33	SR: 26; DA: 12	mTICI 0-2a	2.7 (0.8)	Solitaire: 74; Enterprise: 3; Wingspan: 1
Sajja et al., 2022 [13]	Retrospective, MC	26	54	27	18 [14-23]	-	12	-	mTICI 0-2a	2 [2-3]	Promus: 3; Xience Xpedition: 7; Xience V: 6; Xience Prime: 1; Liberte: 1; Bare: 3; Resolute Onyx: 3
Yi et al., 2021 [15]	Retrospective, SC	31	66 (12.9)	36	4.5 [1-14]	-	29	SR: 31	Insufficient distal flow restoration	-	Neuroform: 31
Stracke et al., 2020 [14]	Retrospective, MC	210	67 [59-75]	40	13 [8-18]	9 [8-10]	31	SR: 210	Acute reocclusion/persistent high-grade stenosis	2 [1-3]	Solitaire: 45; Acclino: 61; Wingspan:8; Neuroform: 65; others: 31
Meyer et al., 2020 [16]	Retrospective, MC	41	65 [48-73]	29	7 [4-8]	8 [8-10]	61	SR: 41	mTICI 0-2a	2 [1-4]	Acclino flex, Neuroform, Solitaire, Enterprise, Wingspan
Dobrocky et al., 2019 [43]	Retrospective, SC	10	71 [67-79]	30	11 [9-15]	-	30	SR: 10	Persistent severe intracranial stenosis >70%	1.5 [1-3]	Wingspan: 9; Xience: 1
Feng et al., 2019 [35]	Retrospective, SC	55	65 (9)	9	12.5	-	36	SR: 55	-	-	Solitaire: 15; Apollo: 13; Enterprise: 27
Li D-D et al., 2019 [36]	Retrospective, SC	39	68 (43-91)	46	22 [9-36]	8 [6-10]	31	SR: 39	mTICI 0-2a	-	Solitaire: 39
Woo et al., 2018 [47]	Retrospective, SC	27	-	44	-	-	50	SR: 27	Refractory occlusion	-	Solitaire: 13; other; 14
Nappini et al., 2018 [45]	Retrospective, SC	17	62	41	20 (9-35)	9 (7-10)	47	SR: 17	mTICI 0-2a	4	Solitaire: 17
Delgado-Acosta et al., 2017 [42]	Retrospective, SC	42	61 [53-72]	29	-	-	24	-	-	-	Enterprise: 42
Forbrig et al., 2018 [44]	Retrospective, MC	34	67	32	13 (4-26)	-	32	SR: 34	Immediate reocclusion or remaining high grade stenosis	2	Solitaire: 7; Acclino flex: 6; Enterprise: 15; Wingspan: 6
Linfante et al., 2011 [37]	Retrospective, SC	19	64.9 (15.2)	16	19	-	-	-	Minimal or no revascularization	-	Enterprise: 6; Wingspan: 13
Sauvageau et al., 2007 [46]	Retrospective, SC	10	73 (35-93)	-	16 [14-19]	-	-	SR: 10	mTICI 0-1	-	Neuroform: 6; Vision: 3; MiniVision: 1

NIHSS: National Institutes of Health Stroke Scale; ASPECTS: Alberta Stroke Program Early Computed Tomography Score; IV-tPA: intravenous tissue plasminogen activator; MT: Mechanical thrombectomy; RS: Rescue stenting; MC: Multicentric; SC" Single-center; SR: Stent-retriever; DA: Direct aspiration; mTICI: modified Thrombolysis in Cerebral Infarction.

Supplementary Table 4. Additional characteristics.

Study	Period of study inclusion	Location		Onset to groin (hours)*	
		RS	MM	RS	MM
Tschoe et al., 2022	Jan 2014 - Jun 2019	ICA: 12; M1: 29; M2: 7; A1/A2: 1	ICA: 75; M1: 165; M2: 21; A1/A2: 2	-	-
Sweid et al., 2022	Jan 2010 - Oct 2019	Anterior: 26	Anterior: 76	8.3 (5.5)	9.3 (8.9)
Mohammaden et al., 2022	Jan 2015 - Dec 2020	ICA: 64; M1: 159; M2: 30	ICA: 66; M1: 99; M2: 81	8.6 [4.2-15.4]	5.1 [3.3-9.9]
Luo et al., 2021	-	-	-	4.3	4.8
Hassan et al., 2021	Dec 2012 - Aug 2020	-	-	-	-
Perez-Garcia et al., 2020	Sep 2009 - Apr 2019	ICA: 9; M1: 11	ICA: 12; M1: 28	5.2 (4.8)	3.7 (1.2)
Peng et al., 2020	Jan 2015 - Jun 2018	-	-	4.9	4.9
Cornelissen et al., 2019	Oct 2013 - Mar 2017	ICA: 1; M1: 8; Basilar: 2	M1: 9; M2: 1; Basilar: 4	4.8 (2.5)	4.5 (2.9)
Zhou et al., 2018	Jan 2014 - Dec 2016	-	-	-	-
Baracchini et al., 2017	Jan 2014 - Jul 2016	ICA: 2; MCA: 18	ICA: 2; M1: 19	3.4 (0.9)	3.3 (0.8)
Chang et al., 2018	Sep 2010 - Dec 2015	-	-	3.9 (2)	4.2 (1.8)
Baek et al., 2021	Jan 2010 - Dec 2018	ICA: 5; MCA: 20	ICA: 9; MCA: 15	4.3 [3.7-6.3]	4.8 [3-6.9]
Baek et al., 2022	Jun 2019 - Mar 2021	ICA: 12; MCA: 66	-	5	-
Sajja et al., 2022	Dec 2015 - Jan 2021	ICA: 6; MCA: 12; Basilar 7	-	-	-
Yi et al., 2021	Jun 2018 - Dec 2019	ICA: 5; MCA: 18; Basilar: 8	-	-	-
Starcke et al., 2020	Feb 2012 - Oct 2018	ICA: 41; M1: 85; M2: 8; A1/A2: 1; Basilar 46	-	-	-
Meyer et al., 2020	Jan 2014 - Dec 2018	ICA: 15; M1: 22; M2: 4	-	-	-
Dobrocky et al., 2019	Feb 2010 - Aug 2017	MCA: 10	-	-	-
Feng et al., 2019	May 2013 - Feb 2018	Anterior: 35	-	3.5	-
Li D-D et al., 2019	Aug 2014 - May 2018	ICA: 21; MCA: 13; Basilar: 5	-	4.8	-
Woo et al., 2018	Jan 2011 - Jan 2016	ICA: 10; M1: 12; M2: 1; Basilar: 4	-	-	-
Nappini et al., 2018	Jul 2010 - Feb 2016	ICA: 10; MCA: 7	-	3.9	-
Delgado-Acosta et al., 2017	Dec 2008 - Jan 2016	ICA: 14; M1: 15; Basilar: 13	-	-	-
Forbrig et al., 2018	Nov 2012 - Aug 2017	Anterior: 18; Basilar: 12	-	-	-
Linfante et al., 2011	Aug 2008 - Sept 2010	ICA: 6; M1: 10; Basilar: 3	-	-	-

Sauvageau et al., 2007	Feb 2005 - May 2006	M1: 10	-	-	-

RS: Rescue stenting; MM: Medical management; ICA: Internal carotid artery; MCA: Middle cerebral artery; M1: M1 segment of the middle cerebral artery; M2: M2 segment of the middle cerebral artery.

* Mean (Standard deviation); Median [Interquartile range]

Supplementary Table 5. Antiplatelet regimens.

Study	Intraprocedural	Postprocedural
Tschoe et al., 2022	IV GP IIbIIIa bolus prior stent placement followed by oral DAPT load within 6 hours or IV GP IIbIIIa bolus prior stent placement with subsequent infusion for up to 24 hours followed by oral DAPT or oral load only of DAPT.	-
Sweid et al., 2022	Tirofiban was administered as an IV bolus of 25 mcg/kg before the stent placement followed by a second oral antiplatelet agent. The second antiplatelet agent includes a loading dose: 300/600 mg of Clopidogrel or 120/180 mg of Ticagrelor.	-
Mohammaden et al., 2022	-	-
Luo et al., 2021	Tirofiban is initiated with a loading dose of 0.4 μg/kg/min over 30min followed by a continuous infusion of 0.1 μg/kg/min over 24 h. DAPT with 100mg ASA and 75mg clopidogrel was started either orally or via a nasogastric tube 4 h before stopping the infusion of tirofiban.	-
Hassan et al., 2021	Tirofiban bolus 12mcg/kg over 30 min followed by infusion rate of 0.1mcg/kg/min (ml/h).	-
Perez-Garcia et al., 2020	900 mg IV lysine acetylsalicylate (equivalent to 500 mg ASA) associated with abciximab or tirofiban IV bolus (the latter followed by continuous perfusion for 12–24 h) previous to stent implantation.	-
Peng et al., 2020	-	-
Cornelissen et al., 2019	Half or full bolus dose of weight-adapted abciximab or ASA was given IV. An IV abciximab infusion was not routinely used.	If there were no contraindications on the imaging studies, DAPT of ASA in combination with either clopidogrel or prasugrel was commenced. This was continued for 3–6 months and subsequently the patients were de-escalated to 100mg ASA for life.
Zhou et al., 2018	IV tirofiban 8.0 g/kg bolus over a period of 3 minutes immediately before stent deployment, followed by a maintenance dose of 0.10 g/kg/min up to 24 hours after procedure.	Two hours before the cessation of tirofiban, dual antiplatelet drug therapy with clopidogrel (300 mg) and aspirin (300 mg) was overlapped with one-half the dose of the IV infusion, followed by a dosage of 75 mg clopidogrel and 100 mg aspirin daily for 3-6 months.
Baracchini et al., 2017	During the procedure, if not yet administered, an IA bolus (25 mg/kg in 3 minutes) of Tirofiban was injected, followed by 12 hours IV infusion (0.1 mg/kg).	DAPT for 3 months.
Chang et al., 2018	-	-
Baek et al., 2021	(1) IV infusion of GPI after completion of the EVT procedure for at least 12 hours.<br>(2) None.	No antithrombotic medication until intracranial hemorrhage was excluded based on brain imaging on the next day of EVT.<br>(1) Then administration of DAPT after exclusion of intracranial hemorrhage based on brain imaging on the next day of EVT.<br>(2) Administration of single (ASA 100–300 mg or clopidogrel 75 mg) or DAPT (ASA 100–300 mg with clopidogrel 75 mg) immediately after completion of the EVT procedure.
Baek et al., 2022	0.2 to 1.5 mg of tirofiban (0.05 mg/mL concentration with 0.1 mg/min) for IA infusion. For IV infusion of tirofiban, the total dose and infusion rate were calculated based on the patient's body weight according to pharmacopeial guidance (usually 0.1 μg/kg/min).	Postprocedural antiplatelet medication included (1) IV infusion of tirofiban for 12 to 24 hours after EVT, followed by administration of oral DAPT (ASA 100 mg with clopidogrel 75 mg); and (2) immediate administration of oral DAPT (ASA 100 mg with clopidogrel 75 mg; or by its loading dose if necessary) after completion of the EVT.
Sajja et al., 2022	DAPT load and a continuous infusion of IV tirofiban with or without a bolus while the oral antiplatelet medications reach a therapeutic target. For patients who received IV tPA, some of the operators used IV tirofiban as a single bolus dose without the infusion to avoid intracranial bleeding complications. Many of the operators also started preferring Ticagrelor as opposed to Clopidogrel as the second antiplatelet agent in addition to ASA.	-

Yi et al., 2021	IA tirofiban over 5 minutes (0.05 mg/min) before stenting or a loading dose of DAPT medications (300 mg aspirin and 300 mg clopidogrel) were applied in patients who have not used IA tirofiban, immediately before the balloon angioplasty and intracranial stenting.	-
Starcke et al., 2020	-	-
Meyer et al., 2020	IV ASA only or GP IIbIIIa inhibitor.	DAPT for 3 months.
Dobrocky et al., 2019	If thrombus formation occurred within the stent, IA abciximab was slowly injected.	-
Feng et al., 2019	IV GP IIb/IIIa inhibitor was administered according to the patient's body weight and maintained by micropump. After the patient was awake or the gastric tube was placed, a loading dose of antiplatelet drug (aspirin 300 mg, clopidogrel 300 mg) was given. The glycoprotein IIb/IIIa inhibitor continues to maintain the micropump for 24-36 hours.	ASA 100 mg and clopidogrel 75 mg once daily for 3 months after the procedure. If the patient still has a stroke after surgery, we adjust the antiplatelet treatment plan, such as changing clopidogrel to ticagrelor or increasing the dose of aspirin according to the test results of thromboelastic map. Clopidogrel is stopped after 3 months, and patients continue taking aspirin 100 mg once daily for a lifetime.
Li D-D et al., 2019	During the temporary deployment of the Solitaire stent, adjuvant tirofiban was injected at 0.2mg/kg/minute through a microcatheter holding a Solitaire stent for 30 minutes.	Patients underwent CT scanning immediately after the procedure to exclude cerebral hemorrhage. If the postprocedural CT demonstrated no cerebral hemorrhage, IV tirofiban administration was continued at a rate of 0.1 mg/kg/minute for up to 24 hours. DAPT (75 mg clopidogrel and 100 mg ASA) was provided during the last 6 hours of tirofiban administration and was maintained for 3 months.
Woo et al., 2018	IV Tirofiban was administered if the patient was about to undergo permanent stent insertion and had not taken proper antithrombotic medication before the procedure. Tirofiban was initially injected as a loading dose (0.4 mcg/kg) for 30 minutes, followed by continuous infusion for prevention of acute in-stent thrombosis (0.1mcg/kg/min).	The patients immediately received additional oral ASA (300 mg) and clopidogrel (75 mg) to protect the stent after excluding symptomatic hemorrhagic transformation and/or brain edema with significant mass effect with brain CT on the following day.
Nappini et al., 2018	After stent detachment, an IV bolus of Tirofiban was administered according to the weight (25 mcg/kg) in 3 minutes, followed by a 12-hours IV infusion (0.1 mcg/kg).	After 12 hours CT was performed to rule-out asymptomatic or sICH and, if no bleeding was reported, ASA 300mg and loading dose of clopidogrel (300 mg) were administered. DAPT with ASA 100mg and clopidogrel 75mg was continued for 3 months followed by a lifelong single antiplatelet treatment with ASA.
Delgado-Acosta et al., 2017	IV abciximab 0.1 mg/kg	-
Forbrig et al., 2018	Tirofiban: as recommended by the manufacturer starting with a weight adapted IV loading dose of 0.4 µg/kg/min for 30min, followed by a maintenance dose of 0.1 µg/kg/min IV for at least 12h. Overlapping, DAPT with 100mg ASA and 300mg clopidogrel was started either orally or via the gastric tube 4h prior to infusion stop.	If the routine control CT within the following day showed major sICH, the antiplatelet therapy was stopped. Otherwise, it was continued orally with ASA 100mg/day life long and clopidogrel 75mg/day for at least 6 months.
Linfante et al., 2011	If the patient was not using aspirin and clopidogrel, then IA abciximab was infused	-
Sauvageau et al., 2007	A single IA or IV bolus dose of eptifibatide (180 µg/kg body weight) was administered immediately before stenting, without a maintenance infusion.	ASA (325–650 mg) and clopidogrel (300–600 mg) were administered immediately after the procedure. A DAPT regimen of 75 mg clopidogrel daily and 325 mg ASA daily was maintained for 6 weeks after the procedure, after which patients were to remain on 325 mg daily ASA therapy indefinitely.

IV: Intravenous; IA: Intraarterial; GPI: Glycoprotein inhibitor; DAPT: Dual antiplatelet therapy; EVT: Endovascular treatment; CT: Computed tomography; sICH: Symptomatic intracerebral hemorrhage.

Supplementary Table 6. Assessment of publication bias using the Egger's test for studies including each outcome.

	**p-value**
Favorable functional outcome	0.3179
Successful reperfusion	Not calculated
sICH	0.6257
90-day mortality	0.3346

sICH: symptomatic intracranial hemorrhage

Supplementary Table 7. Sensitivity analyses.

Study selection criteria	Outcome	No. of studies	No. of participants	OR (95% CI)	I^2
Excluding studies with a low rate (<40%) of IV-tPA in the control arm	Favorable functional outcome	7	767	2.81 (1.94 – 4.08)	0%
Fixed-effect model	Favorable functional outcome	12	1773	3.17 (2.48 – 4.04)	64%
Excluding studies with a definition of failed MT not based on the initial mTICI	Successful reperfusion	16	961	85% (80 – 89%)*	67%
Studies with stent-retriever as the exclusive first-line MT technique before RS	sICH	4	472	0.65 (0.35 – 1.21)	0%
Excluding studies with a low rate (<40%) of IV-tPA in the control arm	Mortality at 90 days	6	678	0.42 (0.17 – 1.03)	61%

No.: number; OR: odds ratio; CI: Confidence interval; sICH: Symptomatic intracranial hemorrhage; MT: Mechanical thrombectomy; RS: Rescue stenting; IV-tPA: Intravenous tissue plasminogen activator; mTICI: modified thrombolysis in cerebral infarction; IV-tPA: intravenous tissue plasminogen activator;
*Pooled rate.

Supplementary Table 8. Comparative meta-analyses of rescue stenting.

Study	Design	Definition of failed MT	N studies	N participants		RS vs. Control (%); pooled odds ratio [95% CI]		
				RS	Control	90-day mRS 0-2	Final mTICI 2b-3	sICH
Almallouhi et al., 2023 [24]	C	Intracranial atherosclerosis large-vessel occlusion	7	352	155	3.19 [1.91–5.32]	5.05 [2.28–11.22]	0.80 [0.35–1.81]
Cai et al., 2023 [22]	C	mTICI ≤ 2b	7	354	350	41% vs 15%;<br>3.96 [2.69–5.84]	82% (80%–85%)*	8.2% vs. 15.4%;<br>0.64 [0.39–1.04]
Premat et al., 2020 [11]	C	At least one MT attempt in patients with refractory occlusions	4	149	203	43% vs. 21%;<br>2.87 [1.77–4.66]	-	12% vs. 18%;<br>0.68 [0.37–1.27]
Current study	C	According to each study definition	12	691	1082	41% vs. 21%;<br>3.27 [2.08–5.16]	87% (82%–91%)*	9% vs. 12%;<br>0.85 [0.59–1.20]

C: Comparative; RS: Rescue stenting; CI: Confidence interval; mRS: Modified Rankin scale; mTICI: modified Thrombolysis In Cerebral Infarction; sICH: Symptomatic intracranial hemorrhage; MT: Mechanical thrombectomy; N: Number.
*Pooled rates (95% CI)

SUPPLEMENTARY FIGURES

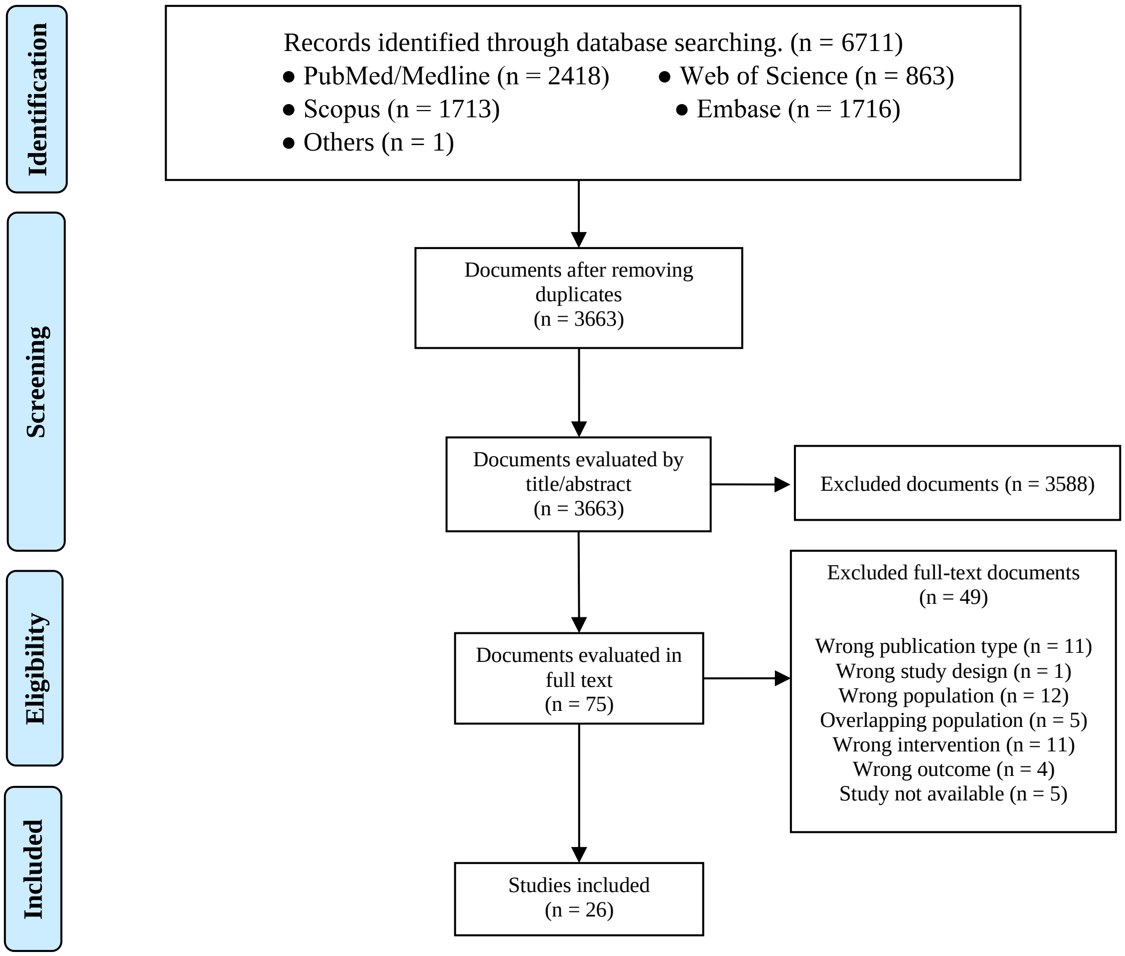

Supplementary Figure 1. Flow chart of study selection.

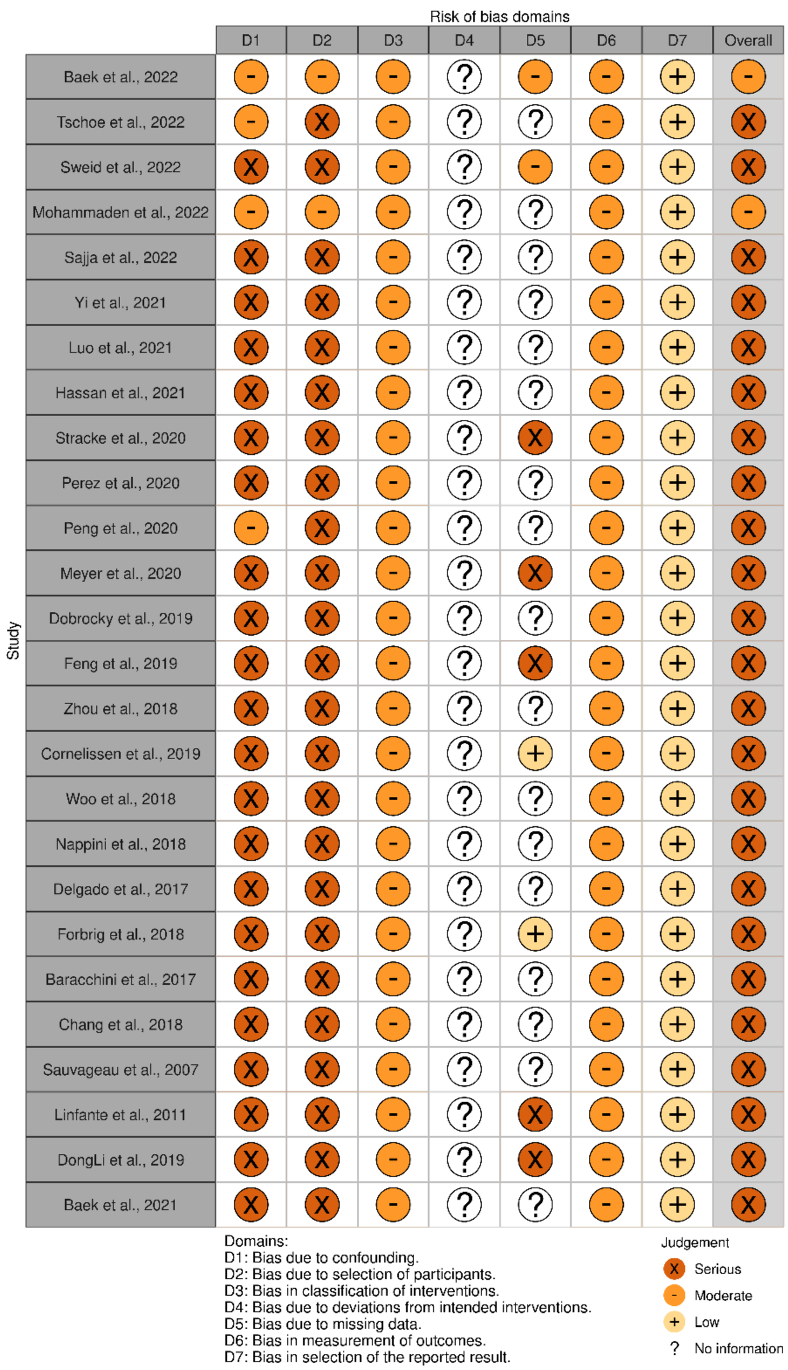

Supplementary Figure 2. Quality assessment of the studies included (according to the Risk of Bias In Non-randomized Studies of Interventions [ROBINS-I] for non-randomized studies).

A

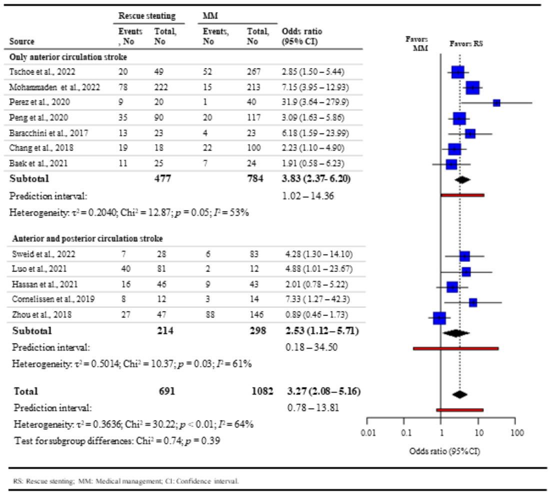

B

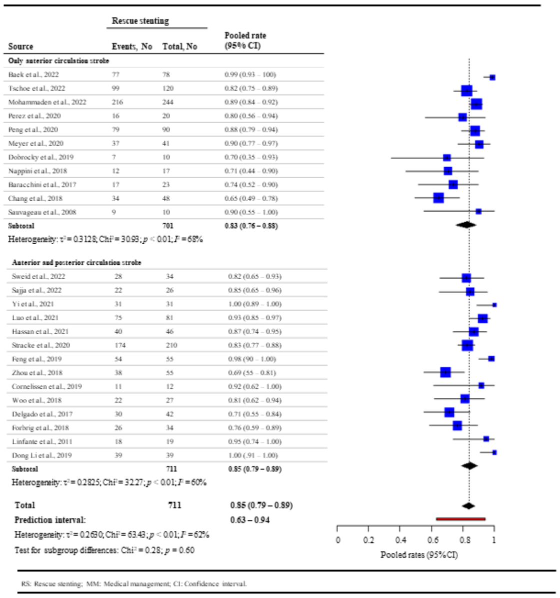

C

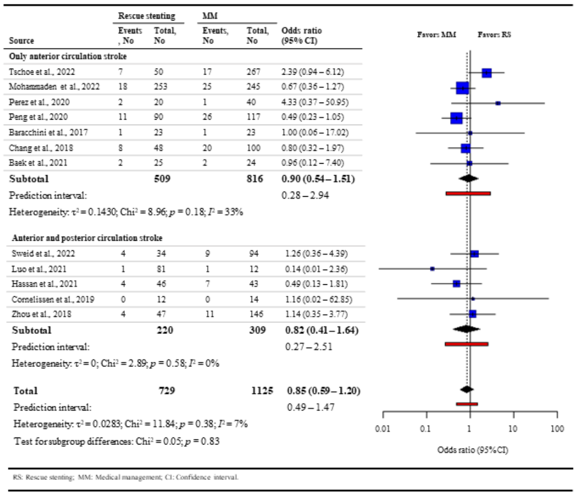

D

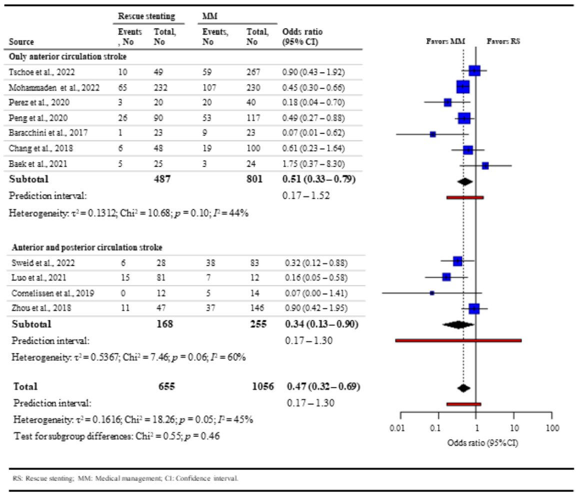

Supplementary Figure 3. Subgroup analysis by the-stroke territory included for (A) favorable functional outcome, (B) successful reperfusion, (C) symptomatic intracranial hemorrhage, and (D) mortality at 90 days.

MM: medical management; CI: confidence interval

A

Source	Rescue stenting Events, No	Rescue stenting Total, No	MM Events, No	MM Total, No	Odds ratio (95% CI)
**Studies with similar stroke severity between arms**					
Tschoe et al., 2022	20	49	52	267	2.85 (1.50 – 5.44)
Hassan et al., 2021	16	46	9	43	2.01 (0.78 – 5.22)
Perez et al., 2020	9	20	1	40	31.91 (3.64 – 279.9)
Cornelissen et al., 2019	8	12	3	14	7.33 (1.27 – 42.29)
Zhou et al., 2018	27	47	88	146	0.89 (0.46 – 1.73)
Baracchini et al., 2017	13	23	4	23	6.18 (1.59 – 23.99)
Chang et al., 2018	19	48	22	100	2.32 (.10 – 4.90)
Baek et al., 2021	11	25	7	24	1.91 (0.58 – 6.23)
**Subtotal**		**270**		**657**	**2.69 (1.5 – 4.75)**
Prediction interval:					0.52 – 13.91
Heterogeneity: $\tau^2 = 0.3668$; $Chi^2 = 17.71$; $p = 0.01$; $I^2 = 60\%$					
**Studies with different stroke severity between arms**					
Sweid et al., 2022	7	28	6	83	4.28 (1.30 – 14.10)
Mohammaden et al., 2022	78	222	15	213	7.15 (3.95 – 12.93)
Luo et al., 2021	40	81	2	12	4.88 (1.01 – 23.67)
Peng et al.,	35	90	20	117	3.09 (1.63 – 5.86)
**Subtotal**		**421**		**425**	**4.74 (3.0 – 7.49)**
Prediction interval:					1.26 – 17.79
Heterogeneity: $\tau^2 = 0.0399$; $Chi^2 = 3.63$; $p = 0.30$; $I^2 = 17\%$					
**Total**		**691**		**1082**	**3.27 (2.1 – 5.16)**
Prediction interval:					0.78 – 13.81
Heterogeneity: $\tau^2 = 0.3636$; $Chi^2 = 30.22$; $p < 0.01$; $I^2 = 64\%$					
Test for subgroup differences: $Chi^2 = 2.31$; $p = 0.13$					

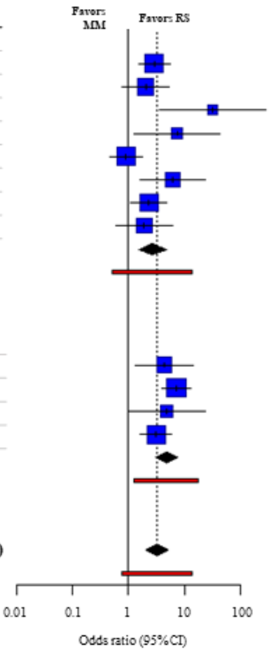

RS: Rescue stenting; MM: Medical management; CI: Confidence interval.

B

Source	Rescue stenting Events, No	Rescue stenting Total, No	MM Events, No	MM Total, No	Odds ratio (95% CI)
**Studies with similar stroke severity between arms**					
Tschoe et al., 2022	10	49	59	267	0.90 (0.43 – 1.92)
Perez et al., 2020	3	20	20	40	0.18 (0.04 – 0.70)
Cornelissen et al., 2019	0	12	5	14	0.07 (0.0 – 1.41)
Zhou et al., 2018	11	47	37	146	0.90 (0.42 – 1.95)
Baracchini et al., 2017	1	23	9	23	0.07 (0.01 -0.62)
Chang et al., 2018	6	48	19	100	0.61 (0.23 – 1.64)
Baek et al., 2021	5	25	3	24	1.75 (0.37 – 8.30)
**Subtotal**		**224**		**614**	**0.54 (0.27 – 1.06)**
Prediction interval:					0.08 – 3.44
Heterogeneity: $\tau^2 = 0.4010$; $Chi^2 = 12.81$; $p = 0.05$; $I^2 = 53\%$					
**Studies with different stroke severity between arms**					
Sweid et al., 2022	6	28	38	83	0.32 (0.12 – 0.88)
Mohammaden et al., 2022	65	232	107	230	0.45 (0.30 – 0.66)
Luo et al., 2021	15	81	7	12	0.16 (0.05 – 0.58)
Peng et al., 2020	26	90	53	117	0.49 (0.27 – 0.88)
**Subtotal**		**431**		**442**	**0.42 (0.31 – 0.57)**
Prediction interval:					0.22 – 0.81
Heterogeneity: $\tau^2 = 0.1616$; $Chi^2 = 2.77$; $p = 0.43$; $I^2 = 0\%$					
**Total**		**655**		**1056**	**0.47 (0.32 – 0.69)**
Prediction interval:					0.17 – 1.30
Heterogeneity: $\tau^2 = 0.1616$; $Chi^2 = 18.26$; $p = 0.05$; $I^2 = 45\%$					
Test for subgroup differences: $Chi^2 = 0.40$; $p = 0.53$					

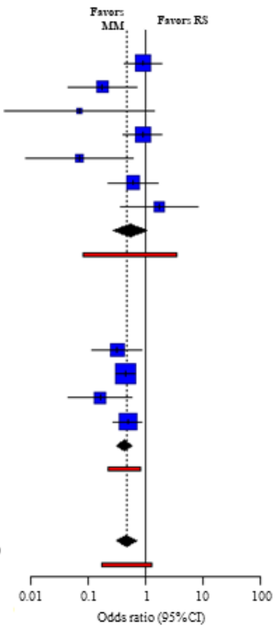

RS: Rescue stenting; MM: Medical management; CI: Confidence interval.

Supplementary Figure 4. Subgroup analysis by studies with similar versus different stroke severity between arms for (A) favorable functional outcome, (B) successful reperfusion, (C) symptomatic intracranial hemorrhage, and (D) mortality at 90 days.

MM: medical management; CI: confidence interval
